# The Impact of Coffee, Matcha, Protein Drinks, and Water Storage on the Microhardness and Color Stability of a Nano-Ceramic Hybrid Composite CAD/CAM Blank

**DOI:** 10.3390/jfb16120444

**Published:** 2025-11-28

**Authors:** Hanin E. Yeslam, Atheer Alkhaldi, Ghadah Alshehri, Maher S. Hajjaj

**Affiliations:** 1 Department of Restorative Dentistry, Faculty of Dentistry, King Abdulaziz University, Jeddah 21589, Saudi Arabia; mhajjaj@kau.edu.sa; 2 Advanced Technology Dental Research Laboratory, Faculty of Dentistry, King Abdulaziz University, Jeddah 21589, Saudi Arabia; dent.atheer@gmail.com (A.A.); ghadah.alshehri99@gmail.com (G.A.); 3 Faculty of Dentistry, King Abdulaziz University, Jeddah 21589, Saudi Arabia

**Keywords:** CAD/CAM, glass ceramics, esthetic dentistry, restorative dentistry, nano-ceramic hybrid composite, biomimetic restorations, microhardness

## Abstract

This study aimed to evaluate the effect of popular beverages, coffee, matcha, and protein isolate, on the microhardness and color stability of feldspar glass ceramic (VB) and nano-ceramic hybrid composite (GD) CAD/CAM materials. Three hundred specimens were prepared and divided into control and immersion groups (water, coffee, matcha, protein). Vicker’s microhardness (HN) was recorded for the control group and post-immersion groups, while color changes were measured before and after immersion. Microhardness values (HN) and color change (ΔE00) were statistically analyzed using the Kruskal–Wallis test followed by Dunn’s post hoc test (*p* < 0.05). Results: The HN values of all VB and GD immersion subgroups were significantly lower than those of the control groups (*p* < 0.001). The VB water immersion group had a significantly lower HN than the protein and matcha immersion groups. The GD immersion groups showed no significant difference in HN between them (*p* > 0.05). VB had a significantly lower ΔE00 (>3.5) and higher HN (790.8 ± 123.62 kgf/mm^2^) than GD (175.22 ± 28.95 kgf/mm^2^) (*p* < 0.001). Coffee caused the greatest ΔE00 in both VB and GD, whereas protein caused the lowest ΔE00 in GD. Conclusion: The study revealed that the feldspar glass ceramic CAD/CAM material had higher microhardness and color stability than the nano-ceramic hybrid composite. Immersion reduces the microhardness and color stability of CAD/CAM ceramics. Matcha and protein have less impact on glass ceramic microhardness, with protein causing less discoloration in nano-ceramic hybrid composites than other immersion media.

## 1. Introduction

Computer-assisted design/computer-assisted manufacturing (CAD/CAM) technology gained popularity for its ability to quickly and accurately produce indirect restorations [1,2]. Dental restorative materials have gradually evolved from metallic alloys to tooth-colored ceramics and resins [3,4]. Previous literature indicates that CAD/CAM restorations perform better than traditional lab-made ones in esthetics, longevity, and marginal accuracy [5,6,7,8]. CAD/CAM restorative materials are broadly categorized into exceptionally strong zirconia polycrystalline ceramics, high-strength glass-ceramics, including feldspar, leucite-reinforced, and lithium disilicate ceramics that are valued for their esthetics and durability, and nanoceramic hybrids, including resin nanoceramics and resin-infiltrated ceramic network materials that combine ceramic and resin systems to offer superior machinability and fracture resistance [9,10]. CAD/CAM materials that do not require post-milling sintering are increasingly popular due to their convenience and practicality [11]. Materials like ceramic hybrid composites and feldspar glass ceramics can restore missing tooth structure, matching the esthetics and mechanical properties of natural tooth structure [12]. Indirect (CAD/CAM) composite materials, an alternative to ceramic blocks, offer easier repair and maintenance, and cause less wear to the opposing tooth surface [13]. Compared to direct composites, indirect ceramic hybrid composite materials have less polymerization shrinkage, porosity, better homogeneity, and color stability [14]. Nano-ceramic hybrid is a relatively new material that has been reported for its biocompatibility and good mechanical and esthetic properties that mimic natural teeth [15,16,17]. CAD/CAM ceramics match the microhardness (MH) values of tooth enamel, which has a microhardness value ranging from 2.23 to 7.18 GPa, while dentin ranges from 0.71 to 0.92 GPa [18,19].

Dental restorative materials, including composites and ceramics, must withstand intraoral occlusal forces and maintain good color stability for long-term survival [3,7]. Microhardness, which indicates the surface’s resistance to indentation or penetration, is a crucial consideration in various industries and is used to determine the durability of dental restorative material [18,20]. The resin matrix composition of nano-ceramic hybrid materials can influence the surface microhardness and staining of the milled restoration [21]. Therefore, these ceramic hybrids may respond differently to various intraoral challenges compared to other CAD/CAM ceramics. Eating habits worldwide have become more complex with the introduction of various supplements and processed foods, such as protein drinks, to help achieve optimal daily intake of minerals and proteins [22]. Matcha, a Japanese ground green tea beverage, has gained significant traction worldwide due to its therapeutic effects [23]. Currently, the impact of these foods and drinks on the microhardness and color stability of dental materials remains to be explored.

The consumption of beverages with varying acidities and temperatures, such as coffee, tea, soft drinks, and protein shakes, may affect the physical, mechanical, and esthetic properties of teeth and restorations [15,24]. The immersion of CAD/CAM lithium disilicate glass ceramics in green tea was found to increase surface roughness, which could indicate a potential negative impact on microhardness [25]. In the same study, coffee and soft drinks were found to produce the highest amount of discoloration in both materials [25]. In another study, protein beverages were found to affect the microhardness of hybrid resin composites [26], but their effect on CAD/CAM ceramic hybrids was not explored. Several acidic beverages, including commonly consumed tea and coffee, can negatively impact the microhardness and color stability of ceramic hybrid, composite, and glass-ceramic CAD/CAM materials [5,27]. Cola drinks and dietary methyl-ethyl ketone (MEK) were also associated with a reduction in the microhardness of CAD/CAM nano-ceramic hybrid composites, compared to zirconia-reinforced lithium silicate glass ceramics [7,28]. Moreover, coffee, wine, and tea significantly reduced the microhardness and color stability of resin, glass-ceramic, and hybrid composite-ceramic CAD/CAM materials, whereas saliva and citric acid had a minimal impact on the resin material [29,30,31,32].

Despite their differences, both materials have similar clinical applications, and the dentist chooses which one to use for treatment planning [33]. Evaluating new generations of nano-ceramic hybrid composite CAD/CAM materials in response to popular beverages, including those with health-promoting properties, could provide valuable information on their hardness, wear resistance, and durability in intraoral performance. Recently, there has been a surge in the popularity of both matcha green tea [34] and protein drinks, such as whey and whey-isolate post-workout protein drinks, which have gained a reputation as superfoods [35]. While research exists on the effects of other popular beverages, such as caffeinated, acidic, carbonated, and energy drinks, on the surface microhardness of dental restorative materials [36,37,38,39,40,41], to the best of the author’s knowledge, none of the studies compared the unique effects of protein and matcha drinks on the surface microhardness and color stability of CAD/CAM ceramic and hybrid composite materials. This lack of comparative data highlighted the need to further study how these popular drinks may affect the performance of indirect restorative materials. This study aimed to compare the microhardness of a nano-ceramic hybrid composite and a glass ceramic CAD/CAM material, intended for the monolithic fabrication of indirect dental restorations, and to evaluate their microhardness response to protein isolate and matcha drinks. The null hypotheses were that there was no difference in the microhardness and color stability of nano-ceramic hybrid composite vs. glass-ceramic CAD/CAM materials and that there was no negative effect caused by matcha, protein isolate, and coffee beverages on the microhardness and color of nano-ceramic hybrid composite CAD/CAM materials.

## 2. Materials and Methods

The current study investigated the microhardness and color stability of two ceramic CAD/CAM materials: a nano-ceramic hybrid composite (Grandio Disc, VOCO GmbH, Cuxhaven, Germany) and a feldspar glass ceramic (Vita Mark II Blocks, Vita Zahnfabrik GmbH, Bad Säckingen, Germany). Table 1 provides detailed information on the composition of each material.

**Figure 1:**
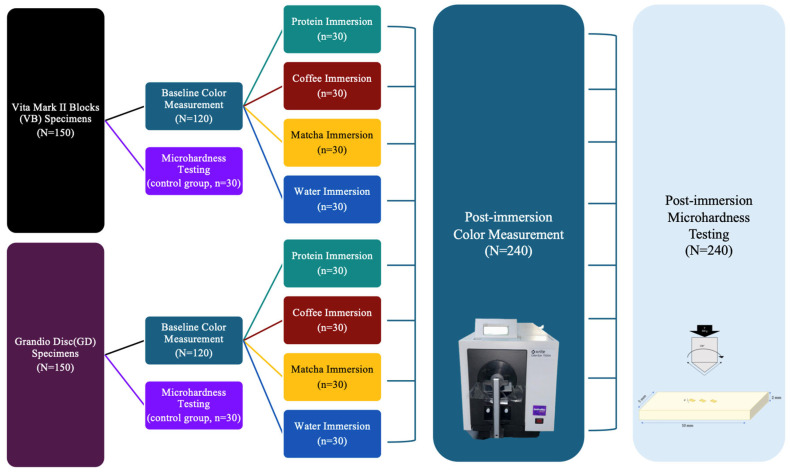
Graphical presentation of the study design.

The fabricated specimens were divided into five groups, four groups corresponding to four different immersion media (water (positive control), coffee, matcha, and protein isolate), and one negative control group with no immersion (for microhardness baseline measurement). Figure 1 provides an overview of the study design.

### 2.1. Specimen Preparation

A total of 300 specimens measuring 10 × 5 × 2 mm^3^ were cut from the CAD/CAM VB blocks and GD disc using a precision low-speed saw cutting machine (TechCut 4^TM^ precision low-speed saw, Allied High-Tech Products, Inc., Rancho Dominguez, CA, USA) using a 100 mm Super-Thin Rim 0.25 mm thick diamond-coated saw blade (Lapidary saw blade Super-Thin Rim, Jingling, China) under constant water irrigation. The thickness of the specimens was 2 mm to mimic the thickest part of a dental crown in clinical scenarios. After cutting, all specimens were visually inspected and examined under 2.5x magnification for fractures and any other visible defects. Specimens with defects were discarded. The finishing and polishing of the specimen sides were performed using silicon carbide (SiC) paper with grits of 600, 800, and 1200, respectively, under continuous irrigation with deionized water. All specimens’ measurements were confirmed using a digital caliper (vernier caliper 200 mm/8 in, Hi-Wendy, New Taipei, Taiwan). Thirty specimens were randomly selected from each tested material to undergo microhardness testing without immersion in any solution as described below in testing procedures (control groups for microhardness). All specimens were stored in distilled water (pH = 7) at 37 °C for 24 h before undergoing control group testing and immersion in their respective solutions.

### 2.2. Immersion Procedures

Thirty specimens from each CAD/CAM material were randomly selected from the remaining untested specimens (N = 240) and grouped to be immersed in one of the following solutions: Protein Isolate (MyProtein MyVegan Clear Vegan Protein, Northwich, Cheshire, UK), Coffee (Nescafé classic, Nestle, Switzerland), Matcha (Kagura, Shizuoka, Japan), and deionized water (positive control group).

The coffee solution was prepared by dissolving 2 g of coffee powder in 200 mL of boiled distilled water, following the manufacturer’s instructions (pH = 4.8). The protein isolate (clear) solution was prepared by mixing one scoop of the powder with 250 mL of deionized water, as recommended by the manufacturer (pH = 5.5). The matcha solution was prepared by sifting 2 g of matcha powder into a bowl, then adding a small amount of 80° C hot water to the powder and whisking it into a smooth paste. 100 mL of hot water was then added, and the solution was whisked vigorously until it became frothy (pH = 5.5). The coffee, protein isolate, water, and matcha solutions were allowed to reach room temperature (25 °C) before immersing the specimen in them. All specimens were stored in their respective staining solutions in an incubator at a temperature of 37 °C for a total of 36 days (24 h/day) as an accelerated aging model to represent 36 months (3 years) of intra-oral use with average daily consumption of 3.2 cups for 15 min per day [44].

Testing Procedures:

- Microhardness:

The specimens from each material’s control group (n = 30) were measured using a Vickers testing machine (ZHV30, Zwick/Roell, Ulm, Germany). The device’s diamond indenter produced three evenly spaced indentations (1 mm apart) on the center of the testing surface of each specimen, using a 200 g heavy load for a duration of 20 s for each indentation.

After immersion in the different media (protein, coffee, matcha, and water as a positive control), all specimens in the immersion groups were subjected to microhardness testing using the same procedure as the control specimen groups.

To determine the Vickers hardness (HV) for each specimen, the average of three readings was used. The microhardness was calculated according to the following formula:

HV = 1.854 P/d^2^

where HV: Vickers hardness in Kgf/mm^2^, P: load in Kgf, and d: mean length of diagonals in mm.

- Color Change:

Color measurement was performed on all specimens in the immersion groups before and then after the 36 day-immersion period. After immersion, the specimens were removed, rinsed gently with deionized water, dried with absorbent paper, and then color measurement was completed before microhardness testing to prevent indent influence on the registered values. Spectrophotometric color measurements were determined for all specimens with a lab-grade desktop spectrophotometer (Color Eye 7000A, X-rite, Grand Rapids, MI, USA) using the CIE (Commission International de L’Eclairage; [45]) L*, a*, and b* parameters, where L* measures lightness (100 is white and zero is black); a* measures redness (positive value) and greenness (negative value); b* measures yellowness (positive value) and blueness (negative value).

Color measurement was performed for each specimen against a white background under the same light source (daylight LED bulb) before and after immersion in the different media by the same investigator to reduce the risk of gross variability. A special jig was used to position the specimens against the spectrophotometer window in a marked location. To standardize color measurement throughout the study, an average of three readings for each specimen was recorded.

The change in L*, a*, and b* color parameters (ΔL, Δa, and Δb) were calculated for each immersion group. The color change (ΔE00) was calculated using the CIEDE2000 (CIE 2001) formula [46]:

ΔE00 = √[(ΔL′/(kL SL))^2^ + (ΔC′/(kC SC))^2^ + (ΔH′/(kH SH))^2^ + RT (ΔC′/(kC SC))(ΔH′/(kH SH))]

where (ΔL′, ΔC′, and ΔH′) represent the differences in lightness, chroma, and hue, respectively, (SL, SC, and SH) represent the weighting functions for lightness, chroma, and hue correction, (kL, kC, and kH) represent the parametric factors set to 1 for reference conditions, and (RT) represents a rotation function accounting for the interaction between chroma and hue differences in the blue region.

### 2.3. Statistical Analysis

For microhardness testing, sample size estimation was done using G*Power 3 software (Version 3.1.9.6, Heinrich-Heine-Universität Düsseldorf, Germany). A study power of 84% to detect a medium effect (f = 0.3) at an alpha error probability of 0.05 would require a total sample size of 150 specimens per ceramic CAD/CAM material and 30 specimens per group (5 groups per CAD/CAM material (n = 30)). For the color change evaluation, a total sample size of 240 specimens would suffice to detect a medium effect between the groups (4 groups per CAD/CAM material (n = 30)) with 80% power at an alpha error probability of 0.05.

The microhardness number (HN) and color change (ΔE00, ΔL, Δa, and Δb) calculations, averages, and descriptive statistics were conducted and tabulated using Microsoft Excel software (Microsoft Office 365, Microsoft Corporation., Redmond, WA, USA). The results data did not follow normal distribution according to the Kolmogorov–Smirnov test (*p* < 0.05); therefore, non-parametric tests were conducted using the DATAtab statistics calculator (DATAtab e.U., Graz, Austria [47]) and R statistical software (R 4.5.1 GUI 1.82 Big Sur Intel build (8536), The R Foundation for Statistical Computing, Vienna, Austria).

Two separate Kruskal–Wallis tests were conducted for microhardness data, one for each ceramic CAD/CAM material, to evaluate the effect of immersion media on the microhardness of each material, taking into account the fundamental differences between them. Dunn-Bonferroni post hoc pairwise comparisons were completed to compare the ceramic microhardness between each pair of immersion media. A Welch’s *t*-test was conducted to compare the HN of the two control groups of GD and VB materials, as the Levene’s test revealed unequal variances for the two groups (*p* < 0.001). All statistical tests were conducted at a significance level of 0.05.

Due to violations of the assumptions of normality (Kolmogorov–Smirnov test) and homogeneity of variances (Levene’s test), a two-way aligned ranks transformation analysis of variance (ART-ANOVA) was conducted to examine the main effects of material type (GD, VB) and immersion medium (protein, coffee, matcha, water), as well as their interaction effect on color change (ΔE00). Dunn’s post hoc test with Holm adjustment was used for multiple comparisons. Effect sizes were reported using Cliff’s Delta for pairwise comparisons and epsilon-squared (ε^2^) for overall effects. Statistical significance was set at α = 0.05.

## 3. Results

### 3.1. Microhardness Results

**Figure 2:**
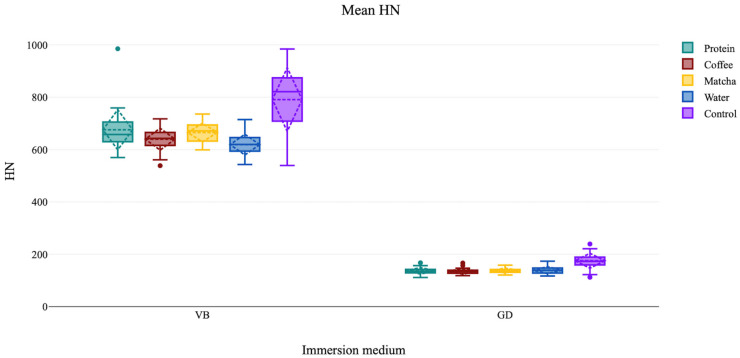
Boxplot diagram showing the mean HN for each ceramic CAD/CAM material in different immersion media.

Within the VB ceramic CAD/CAM material group, the highest microhardness (HN) value was observed in the control, followed by the protein immersion group, then matcha, coffee, and water. In the GD groups, control was the highest, followed by water immersion, which showed the highest HN in the immersion groups, then matcha, protein, and finally coffee. A bar graph showing the mean HN values and standard deviations of the different groups is demonstrated in Figure 2.

In both the VB and GD groups, the Kruskal–Wallis test revealed the presence of a significant difference between the categorical variable immersion medium and the variable HN (*p* < 0.001). Welch’s *t*-test of the two control groups of the VB and GD materials revealed that VB had a statistically significantly higher HN than the GD material (*p* < 0.001).

The Dunn-Bonferroni pairwise comparison test revealed that the control VB had a significantly greater HN than all immersion groups (adj. *p* < 0.05). The VB in water had a statistically significantly lower HN than the VB in protein (adj. *p* = 0.02), and matcha (adj. *p* = 0.01). HN of VB specimens immersed in coffee did not significantly differ than in water (adj. *p* > 0.99). The Dunn-Bonferroni pairwise comparison in the GD material groups showed that control group had a significantly greater HN than all immersed groups (adj. *p* < 0.001), but there was no significant differences between the immersion media groups (adj. *p* > 0.99).

The mean HN for each group, its standard deviation (SD), median, minimum and maximum, and the 95% confidence interval of the mean, as well as the significant statistical differences, are summarized in Table 2.

### 3.2. Color Change Results

**Figure 3:**
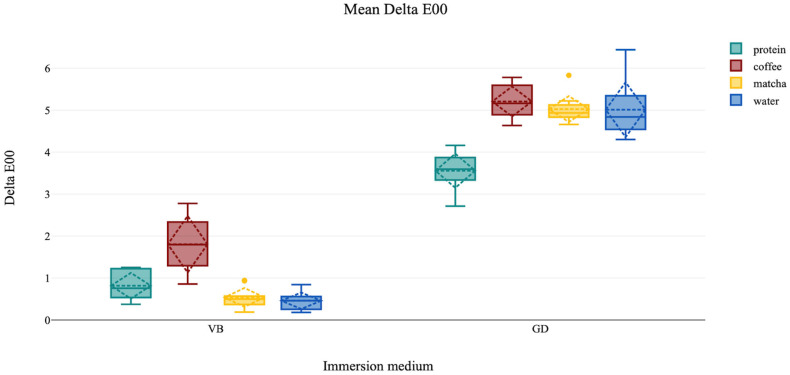
Boxplot diagram showing the mean color change (delta E00) for the glass ceramic (VB) and the nano-ceramic hybrid composite (GD) CAD/CAM material after immersion in the different immersion media.

Within the VB glass ceramic material group, coffee immersion caused the highest color change, followed by protein immersion, then matcha, and water. In the GD nano-ceramic hybrid composite material group, coffee immersion caused the greatest color change, followed by matcha and water immersion, then protein. A bar graph showing the mean color change of the different material/immersion groups is demonstrated in Figure 3.

The ART-ANOVA revealed statistically significant main effects of material (F = 703.26, *p* < 0.001) and immersion medium (F = 103.75, *p* < 0.001), and a statistically significant effect of the interaction between both categorical variables (F = 78.54, *p* < 0.001). Cliff’s Delta effect size for the overall material effect was δ = 0.999, revealing a significantly greater color change in GD specimens than in VB, which was maintained across all immersion groups (GD vs. VB in protein, coffee, matcha, and water; δ = 1.00).

The Dunn’s post hoc test with Holm adjustment revealed that all direct comparisons between GD and VB materials within the same immersion medium were highly significant (mean ε^2^ > 0.22, *p* < 0.001). Within GD material, protein immersion resulted in significantly lower color change compared to coffee (Z = 3.916, ε^2^ = 0.064 *p* = 0.001), matcha (Z = 3.297, ε^2^ = 0.045, *p* = 0.01), and water (Z = −2.879, ε^2^ = 0.035, *p* = 0.036) immersions. Within VB material, coffee immersion produced significantly greater color change compared to matcha (Z = 3.431, ε^2^ = 0.049, *p* = 0.007) and water (Z = 3.95, ε^2^ = 0.065, *p* = 0.001). Coffee caused the greatest decrease in lightness in all groups. Matcha increased the yellowness in GD more than other immersion media, whereas coffee increased the redness in the VB material. The descriptive statistics of color change (ΔE00), changes in color parameters (ΔL, Δa, and Δb), and the statistically significant differences in ΔE00 are summarized in Table 3.

## 4. Discussion

The popularity of health drinks, such as coffee, matcha, and protein, has increased due to the globally rising health awareness, with many consumers opting for these beverages to enhance their nutrition and well-being [26,48]. However, concerns have emerged about their potential effects on dental health, particularly regarding restorative dental materials [37,49]. The current study aimed to investigate the effects of three beverages (coffee, matcha, and protein isolate) on the microhardness and color stability of two ceramic CAD/CAM dental restorative materials, a new nano-ceramic hybrid composite (Grandio Disc, GD) and a feldspar glass ceramic (Vita Mark II, VB) material. The results of this study indicated a significant difference between the tested material groups and between the different immersion media groups (*p* < 0.05). Therefore, the hypotheses that there were no differences in microhardness and color stability between the hybrid composite and ceramic CAD/CAM materials and that immersion in the different media had no negative effects on their microhardness and color stability were rejected.

### 4.1. Discussion of Methodology

In the current study, the choice for the investigated CAD/CAM materials was based on their shared clinical indication, widespread use in clinical practice, and a similar machining method that includes no post-milling firing [50]. Nano-ceramic hybrid composites are relatively new materials aiming to combine the advantages of high strength ceramics with the workability and flexibility of resin materials [51]. The convenience of fast milling, chair-side finishing and polishing, and clinical repairability without the need for further firing increases the appeal of their use by restorative dentists. Ceramic hybrid composite CAD/CAM materials offer superior homogeneity of microstructure compared to direct restorative and 3D printed materials [52], which makes them share several clinical indications with other ceramic CAD/CAM materials, including reinforced varieties, while being less abrasive to opposing dentition [9,33].

Vicker’s microhardness testing was conducted on all material specimens. This test was found to accurately depict the microhardness of thin sections of hard dental restorative materials [7]. It offered a non-destructive testing method; however, cracks could be initiated in brittle materials after testing [53], and such cracks were observed in the pilot VB specimens for this study. Therefore, the pilot specimens were discarded, and separate control material groups were used for baseline microhardness readings to prevent crack-induced variation between specimens. Additionally, the color measurements after immersion in the different media was conducted before final microhardness testing.

Simulation of intra-oral conditions can aid in the evaluation of a material’s wear resistance, material loss, and degradation [26]. Microhardness correlates with a dental material’s strength, degree of conversion, and eventually its intraoral durability. In this study, microhardness changes were assessed to measure the degradation of dental ceramic CAD/CAM materials exposed to coffee, matcha, and protein isolate drinks.

The 24 h per day immersion in different media was designed to simulate one month of beverage consumption, based on an average coffee drinker, as determined in a previous study [54]. According to previous studies, the twenty-four-month clinical success of restorations fabricated from different ceramic CAD/CAM materials was promising and comparable across their different classes [55,56,57]. Therefore, the 36-month-long simulation would provide an insight into the behavior of such materials in response to different dietary challenges at the restorative surface level.

This study used a lab-grade spectrophotometer for color measurement, based on literature to ensure consistency, repeatability, and accuracy, improving reliability [58,59]. The CIEDE2000 (ΔE00) formula was chosen for color change calculation because it is reportedly superior for detecting color changes in dental material and structures. This is due to its ability to correct for visual non-uniformity and compensate for differences in lightness, chroma, and hue [46,60]. Using CIEDE2000 ensures results that are comparable to recent literature and reflects perceptible changes for patients and practitioners [61,62].

### 4.2. Discussion of Microhardness Results

The current study revealed that immersion in various beverages significantly impacted the microhardness of both VB and GD materials. This corresponds with previous studies that demonstrated a deterioration of ceramic CAD/CAM microhardness (both hybrid composite and glass) associated with immersion in different media and different pH solutions [5,17,30].

In the current study, the microhardness of the VB (790.8 ± 123.62 kgf/mm^2^) was significantly greater than that of the GD (175.22 ± 28.95 kgf/mm^2^) control groups, indicating that VB was about 4.5 times harder than GD before exposure to any beverage. This can be attributed to the different chemical composition of the investigated materials and the composite ceramic nature of the GD material, which contains a urethane dimethacrylate (UDMA) resin, making the hybrid composite material less hard than the glassy crystalline structure of VB feldspar ceramic [30,50,63,64]. Feldspar ceramics, such as VB in this study, exhibit a glass phase with densely packed mineral network structure that is fired at a very high temperature, increasing its resistance to indentation [65]. However, this structure also prevents the material from dissipating applied energy through deformation, making glass ceramics more brittle than resin-containing hybrid composites [66].

Following immersion in the different media, the microhardness number of VB remained higher than that of GD in every immersion group. This is in agreement with a recent study by Al Amri et al. comparing VB microhardness to another CAD/CAM ceramic hybrid composite [17]. This also aligned with the different strength and hardness values provided by each material’s manufacturer [42,67]. However, the resultant microhardness values in the control groups of VB and GD were greater than those supplied by the manufacturers (GD: 121.8 kgf/mm^2^; VB: 640 ± 20 kgf/mm^2^) [42,67,68]. This might be partly due to variations in specimen fabrication methods, finishing, polishing, and storage procedures, and differences in exact specimen dimensions compared to the testing performed by the manufacturer.

Dental ceramics are generally considered chemically inert, stable materials, but erosive agents can damage their stability and durability [7]. VB’s reduction in microhardness may be associated with the dissolution of its basic elements, such as silica, aluminum, and potassium, which are released from the glass phase, as reported by Kukiattrakoon et al. [41]. GD’s deterioration by immersion in different media might be caused by hydrolysis and ion leaching from its resinous matrix, as observed in a previous study by Kaur et al. investigating the effect of protein solution on resin composites [26]. This can also be explained partly by the results of Martos et al. [69], where a link between decreased microhardness and the breaking of the siloxane network was reported, triggering surface degradation and softening of the composite resins.

For VB immersion groups, the protein immersion group had the highest microhardness, followed by matcha and coffee. Additionally, immersion in coffee did not elicit a significantly different effect on the microhardness of VB compared to water immersion. However, the water immersion of VB resulted in a significantly lower microhardness than matcha and protein immersion. This might be partly explained by the reported fact that the erosive potential of consumed beverages depends on various factors like their type, concentration, contact time in the mouth, and the buffering capacity of the patient’s saliva [70]. The study results indicated that the presence of protein isolate and matcha may have slowed the dissolution of the ceramic caused by water and/or coffee immersions, which ultimately led to higher microhardness after immersion in protein and matcha compared to water. A recent study found that matcha tea minimally affects elastomeric chains’ strength but changes their color [71], suggesting it might not have a great impact on the mechanical properties of harder dental materials, such as the investigated VB.

Conversely, GD exhibited similar microhardness values across all immersion media, with no significant differences found between the immersion groups. The lack of significant difference in GD microhardness across media aligns with the results of a previous study by Alghamdi et al. that tested a similar ceramic hybrid composite material (Cerasmart (CS), (GC Corporation, Tokyo, Japan)), which demonstrated resistance to hardness loss when immersed in various acidic media [5]. This may suggest a similarity in the high ceramic/polymer ratio (29% by weight in CS and 14% in GD) [72,73], as well as the stability of its polymeric content, as claimed by the manufacturer, which could enhance its mechanical properties. Additionally, the recently reported higher microstructural homogeneity of CAD/CAM ceramic hybrid composite by Prause et al. compared to other hybrid composite materials might also have contributed to GD’s resistance to the harmful effects of immersion in coffee, matcha, and protein isolate, compared to just storage in water [52].

This suggested that while VB is adversely affected by beverage immersion, GD maintained consistent microhardness regardless of the immersion medium, possibly due to its softer resinous component that may have absorbed some of the beverage’s effects.

### 4.3. Discussion of Color Stability Results

The current study demonstrated that the color stability of feldspar glass ceramic (VB) was significantly higher than that of the investigated nano-ceramic hybrid composite (GD), regardless of the immersion condition, which was evident with the almost perfect statistical separation between the color change (ΔE00) values of the two materials and large, clinically significant effect sizes (δ = 1). GD is composed of approximately 86% inorganic fillers, primarily barium aluminium borosilicate glass and silicon dioxide, dispersed within a 14% UDMA/DMA resin [52,67]. Therefore, the greater color change in GD can be attributed to its resin matrix, which is more prone to water sorption and pigment uptake from different beverages and their diffusion within the material [17,25,55,74]. The increased susceptibility of GD to extrinsic discoloration can also be linked to tiny gaps between polymer chains, despite high filler content [75]. In contrast, VB’s fine-structure is comprised mostly of feldspathic crystalline particles embedded within a densely packed inorganic glass matrix (including SiO_2_, Al_2_O_3_, Na_2_O, K_2_O, CaO, and TiO_2_) [42]. This creates a non-porous barrier that prevents the absorption and diffusion of pigments and improves its color stability [11].

To relate the color change (ΔE00) values obtained in this study to clinical relevance, they must be compared against established perceptibility and acceptability thresholds (PT and AT) [11,61]. These thresholds serve as benchmarks in restorative dentistry because not all statistically significant color changes are necessarily clinically relevant [76]. The perceptibility threshold (PT) of ΔE00, which is the smallest color difference detectable by 50% of the observers, usually ranges between 0.8–1.0. While the acceptability threshold (AT) of ΔE00, which is the clinically acceptable level of color difference by both clinicians and patients, usually ranges in the literature between 1.8–2.25 [61]. The ΔE00 in all immersion groups of GD material was greater than both AT and PT, which shows a clinically and socially detectable color change in the material with intraoral use and exposure to various beverages. Whereas only coffee immersion resulted in a color change in VB that is higher than PT and approaching the lower end of the AT range.

The results of the current study showed that immersion media type significantly influenced the ΔE00 of both investigated CAD/CAM materials (*p* < 0.001). Immersion in coffee produced the greatest color change in both VB (ΔE00 = 1.8) and GD materials (ΔE00 = 5.16). Coffee immersion also produced the greatest lightness decrease in both CAD/CAM materials. The increased tendency of coffee to stain materials containing resin is mainly due to the ability of coffee to cause discoloration by surface adsorption and absorption of its yellow chromophores (melanoidins) into the resin organic matrix [77]. The discoloration process in coffee-stained materials can be intensified by the reduced polarity of the coffee solution, which enables the chromophores to permeate deeper into resinous matrices. Research has shown that the pH level of the solution plays a significant role in the degree of discoloration. A pH range of 4–6 has been found to accelerate the discoloration process and considering that coffee typically falls within a pH range of 4.5–5.5, this factor further contributes to the increased discoloration potential [2,17]. Coffee was previously found to be able to stain various resin-containing ceramic hybrid composite materials [78,79], and even a densely packed crystalline lithium disilicate ceramic, which is considered one of the most color-stable ceramics for esthetic dental restorations [25].

On the other hand, immersion in protein isolate, which was of the clear variety, resulted in a significantly lower color change in GD (ΔE00 = 3.59) than immersion in other media, including the positive control immersion in water, suggesting that its staining ability is relatively lower than that of matcha and coffee; however, it was still above the AT and PT. This would indicate its potential for staining even in the absence of the strong colorants of coffee and tea. The matcha (ΔE00 = 0.5) and water as a control (ΔE00 = 0.46) immersion effect was much less pronounced than coffee staining in the VB material, resulting in a color change below PT (<0.8) and AT (<1.8). This color change would probably be considered both imperceptible and clinically acceptable, especially when compared to the coffee-induced staining of VB. This supports the idea that glass ceramics are generally more resistant to staining than resin-containing materials, although coffee remains a potent exception. The lower staining potential of matcha could be related to its lower acidity and tannin content, as well as its catechin and chlorophyll content, which might help prevent adsorption-mediated staining [80].

### 4.4. Clinical Implications, Limitations, and Future Research Directions

The current comparative study showed that the feldspar glass ceramic (VB) had superior microhardness and color stability compared to the nano-ceramic hybrid composite (GD), which could influence dental practitioners’ decisions regarding the CAD/CAM materials to be used in high-esthetic demand cases. Additionally, per the study results, clinicians should advise patients about how commonly consumed beverages, particularly coffee, might impact the surface hardness and esthetic shade of their CAD/CAM restorations. For example, patients who frequently drink protein shakes but less coffee might consider nano-ceramic hybrid composite materials, which tend to discolor the restoration less than coffee. However, clinicians should also consider the lower strength and microhardness of the nano-ceramic hybrid composite material. The current study results emphasize the need for regular evaluation of restorations in patients with high consumption of acidic or intensely colored beverages.

The current study has several limitations that warrant follow-up studies. Firstly, being an in vitro study, it cannot inherently fully replicate all patient-related factors and variations present within the oral cavity, potentially limiting the generalizability of the study findings. This study did not investigate the effect of other beverages and variations in the acidity and temperature of immersion solutions. Further investigation into these factors’ influence on the materials are recommended. The absence of scanning electron microscope imaging of the surface of the investigated material is one of the limitations of the study. Additionally, the timeframe allocated for the study limited the ability to comprehensively assess the long-term color stability of the dental ceramic restorative materials under investigation. Long-term clinical trials and in vivo studies, incorporating other patient variables such as oral hygiene, saliva, and temperature and dietary habits fluctuations, are recommended.

While the study’s focus was on evaluating the effect of common health-promoting beverages on color stability and microhardness, other important mechanical properties such as surface roughness, wear resistance, and flexural strength were not within the scope of the study’s analysis. Future in vitro studies investigating the effects of these beverages on the physical properties of the materials with and without aging simulation are recommended. Lastly, due to the limited number of studies available in the literature examining the impact of protein and matcha beverages specifically on (CAD/CAM) dental restorative materials, performing a comprehensive comparative analysis was challenging. These limitations emphasize the need for further research that aims to understand the multifaceted interactions between these beverages and dental materials in a more comprehensive manner.

## 5. Conclusions

In conclusion:

- The current study demonstrated that the choice of ceramic material for CAD/CAM-fabricated dental restorations might significantly influence their microhardness and color stability, which would therefore consequently impact their durability in response to the consumption of various types of beverages.
- The feldspar glass ceramic (VB) showed superior microhardness and color stability compared to the nano-ceramic hybrid composite (GD), while both materials exhibited varying responses to different immersion media. GD showed good microhardness stability when exposed to various drinks, suggesting its resistance to beverage-induced dissolution.
- Matcha and protein mixed in water might produce a protective effect against glass ceramic material dissolution in aqueous environments; however, further investigation into the specific mechanism and effect on other mechanical properties is warranted.
- Both CAD/CAM materials showed some sensitivity to different immersion media, but the nano-ceramic hybrid composite material maintained consistently high color change values (>3.5) across all conditions. On the other hand, VB glass ceramic material showed more variation in response to different immersion media while maintaining overall superior color stability.
- The study findings highlight the necessity for clinicians to consider the implications of dietary habits on restorative material selection, ensuring that patients receive durable and aesthetically pleasing dental solutions. The findings also reaffirm the need for further research into the long-term effects of various beverages on dental ceramics, as the increasing popularity of health drinks, including matcha and protein beverages, may influence the longevity and performance of dental restorations.

## Figures and Tables

**Table 1:** The composition of the investigated ceramic CAD/CAM materials, as supplied by manufacturers.

Material	Manufacturer	Type	Composition
Vita Mark II Blocks (VB)	Vita Zahnfabrik GmbH, Bad Säckingen, Germany	Fine-structure feldspar glass ceramic blocks	Feldspathic crystalline particles embedded in a glass matrix. (SiO_2_ 56–64%, Al_2_O_3_ 20–23%, Na_2_O 6–9%, K_2_O 6–8%, CaO 0.3–0.6%, TiO_2_ 0.0–0.1%) [42]
Grandio Disc (GD)	VOCO GmbH, Cuxhaven, Germany	86% filled nano- ceramic hybrid composite CAD/CAM disc	86% inorganic fillers: barium aluminium borosilicate glass, silicon dioxide. Resin matrix: 14% UDMA + DMA [43]

Where CAD/CAM is computer-aided design/computer-aided manufacturing, UDMA is Urethane Dimethacrylate, DMA is Dimethacrylate, SiO_2_ is silicone oxide, Al_2_O_3_ is aluminum oxide, Na_2_O is sodium oxide, K_2_O is potassium oxide, CaO is calcium oxide, and TiO_2_ is titanium oxide.

**Table 2:** The descriptive statistics and statistically significant differences for the microhardness values among the tested groups.

Ceramic Material	Immersion Medium	n	Median HN (Kgf/mm^2^)	Mean HN (Kgf/mm^2^)	Std. Dev.	Min.	Max.	95% Confidence Interval (Kgf/mm^2^)
VB	Protein	30	657.5 ^af^	668.01	54.45	569.6	759.9	647.68–688.34
	Coffee	30	642.6 ^e^	639.61	42.47	538.3	717.2	623.75–655.47
	Matcha	30	671.25 ^bd^	665.03	36.14	598.9	735.7	651.53–678.53
	Water	30	619.6 ^abc^	618.8	40.21	542.7	714.6	603.79–633.82
	Control	30	821 ^cdef^	790.8 *	123.62	539	984	744.64–836.96
GD	Protein	30	134.45 ^A^	136.79	12.42	111.2	167.4	132.15–141.43
	Coffee	30	134.4 ^B^	134.9	10.68	118.3	166.5	130.91–138.89
	Matcha	30	135.85 ^C^	137.83	9.18	120.7	158.3	134.4–141.26
	Water	30	138.55 ^D^	139.15	13.14	116.9	173.5	134.24–144.05
	Control	30	173 ^ABCD^	175.22 *	28.95	112	239	164.41–186.03

Where the same lowercase in VB groups superscripts and same uppercase superscripts in the GD groups indicate a statistically significant difference in the Dunn-Bonferroni post hoc tests (*p* < 0.05). The * represents statistically significant difference between the GD and VB materials control groups using the Welch’s *t*-Test (*p* < 0.001).

**Table 3:** The descriptive statistics of color change (ΔE00), changes in color parameters (lightness (ΔL), red-green (Δa), and yellow-blue (Δb) parameters) and statistically significant pairwise differences for ΔE00 values among the tested groups.

	Material	Immersion Medium	n	Median	Mean	Std. Dev.	Q 1	Q 3	95% Confidence Interval for Mean
ΔE00	VB	protein	30	0.76	0.82	0.31	0.54	1.22	0.7–0.93
		coffee	30	1.8 ^AB^	1.8	0.69	1.29	2.33	1.55–2.06
		matcha	30	0.5 ^A^	0.54	0.23	0.37	0.57	0.45–0.62
		water	30	0.46 ^B^	0.46	0.2	0.26	0.56	0.38–0.53
	GD	protein	30	3.59 ^CDE^	3.56	0.41	3.34	3.87	3.4–3.71
		coffee	30	5.16 ^C^	5.21	0.36	4.89	5.59	5.07–5.34
		matcha	30	4.93 ^D^	5.03	0.31	4.83	5.12	4.91–5.14
		water	30	4.84 ^E^	5.01	0.67	4.54	5.34	4.76–5.26
ΔL	VB	protein	30	−0.43	−0.38	0.28	−0.81	−0.18	−0.54–−0.33
		coffee	30	−1.54	−1.53	0.49	−2	−1.21	−1.73–−1.36
		matcha	30	−0.31	−0.34	0.33	−0.48	−0.2	−0.43–−0.19
		water	30	−0.26	−0.22	0.37	−0.56	−0.03	−0.4–−0.12
	GD	protein	30	−3.76	−3.93	0.53	−4.13	−3.39	−3.95–−3.56
		coffee	30	−5.8	−5.72	0.48	−6.3	−5.42	−5.98–−5.62
		matcha	30	−5.37	−5.27	0.35	−5.45	−5.16	−5.5–−5.23
		water	30	−3.78	−4.95	3.81	−5.17	−4.77	−5.21–−2.36
Δa	VB	protein	30	−0.24	−0.25	0.09	−0.32	−0.2	−0.27–−0.21
		coffee	30	0.2	0.21	0.16	0.1	0.33	0.14–0.26
		matcha	30	−0.18	−0.17	0.05	−0.18	−0.15	−0.2–−0.16
		water	30	0	−0.01	0.1	−0.08	0.06	−0.04–0.04
	GD	protein	30	0.76	0.76	0.13	0.64	0.84	0.71–0.81
		coffee	30	1.03	1.07	0.08	0.97	1.1	1.01–1.06
		matcha	30	0.79	0.79	0.1	0.75	0.85	0.75–0.83
		water	30	1.24	1.08	0.56	0.91	1.2	1.03–1.45
Δb	VB	protein	30	0.63	0.57	0.34	0.37	0.96	0.5–0.76
		coffee	30	0.99	0.79	0.66	0.43	1.48	0.75–1.24
		matcha	30	0.07	−0.11	0.34	−0.14	0.19	−0.06–0.19
		water	30	−0.16	−0.18	0.18	−0.25	−0.12	−0.22–−0.09
	GD	protein	30	1.15	1.32	0.45	0.92	1.42	0.98–1.31
		coffee	30	0.47	0.4	0.36	0.29	0.62	0.34–0.61
		matcha	30	1.81	1.97	0.39	1.58	2.06	1.66–1.95
		water	30	1.31	1.3	0.54	0.89	1.69	1.1–1.51

Where the same uppercase superscripts in the ΔE00 median values indicate a statistically significant difference in the Dunn-Holt post hoc tests (*p* < 0.05).

## Data Availability

The data presented in this study are available on request from the corresponding author.
